# Glial contributions to neurodegeneration in tauopathies

**DOI:** 10.1186/s13024-017-0192-x

**Published:** 2017-06-29

**Authors:** Cheryl E. G. Leyns, David M. Holtzman

**Affiliations:** Department of Neurology, Washington University, Hope Center for Neurological Disorders, Knight Alzheimer’s Disease Research Center, 660 S. Euclid Ave, St. Louis, MO 63110 USA

**Keywords:** Tau, Tauopathy, Alzheimer’s disease, Neurodegeneration, Neuroinflammation, Gliosis, Microglia, Astrocyte

## Abstract

Tauopathies are a broad set of neurodegenerative dementias characterized by aggregation of the tau protein into filamentous inclusions that can be found in neurons and glial cells. Activated microglia, astrocytes and elevated levels of proinflammatory molecules are also pathological hallmarks that are found in brain regions affected by tau pathology. There has been abundant research in recent years to understand the role of gliosis and neuroinflammation in neurodegenerative diseases, particularly in Alzheimer’s disease (AD) which is the most common form of dementia. AD is a tauopathy characterized by both extracellular amyloid-β plaques in addition to intracellular neurofibrillary tangles and neuropil threads containing aggregated tau protein. Accumulating evidence suggests that neuroinflammation offers a possible mechanistic link between these pathologies. Additionally, there appears to be a role for neuroinflammation in aggravating tau pathology and neurodegeneration in tauopathies featuring tau deposits as the predominant pathological signature. In this review, we survey the literature regarding inflammatory mechanisms that may impact neurodegeneration in AD and related tauopathies. We consider a physical role for microglia in the spread of tau pathology as well as the non-cell autonomous effects of secreted proinflammatory cytokines, specifically interleukin 1 beta, interleukin 6, tumor necrosis factor alpha and complement proteins. These molecules appear to have direct effects on tau pathophysiology and overall neuronal health. They also indirectly impact neuronal homeostasis by altering glial function. We conclude by proposing a complex role for gliosis and neuroinflammation in accelerating the progression of AD and other tauopathies.

## Background

Abnormal accumulation of the tau protein into intracellular, fibrillar aggregates is observed across a broad spectrum of neurodegenerative disorders that are collectively referred to as tauopathies. Over twenty-five syndromes are currently classified as a tauopathy, which highlights the heterogeneity of these diseases and their vast impact in the dementia field. Primary tauopathies feature tau deposits as the predominant pathological signature and include progressive supranuclear palsy (PSP), corticobasal degeneration (CBD), frontotemporal dementia and parkinsonism linked to chromosome 17 (FTDP-17), Pick’s disease (PiD), chronic traumatic encephalopathy (CTE) and argyrophilic grain disease (AGD) [1, 2]. Alzheimer’s disease (AD), the most prevalent cause of dementia, is categorized as a secondary tauopathy due to the additional presence of amyloid-β (Aβ) plaques and their hypothesized role in initiating AD pathogenesis [3]. In addition to toxic protein aggregates, activated astrocytes and microglia as well as elevated proinflammatory markers are other common pathological hallmarks of tauopathies [4, 5].

The notion that neuroinflammatory processes contribute to disease was once provocative, yet recent studies have uncovered multiple mechanisms by which aberrant gliosis causes detrimental neuroinflammation that can influence tau pathology and accelerate neurodegeneration. One hypothesized mechanism postulates that early activation of microglia stimulates the release of cytokines and chemokines that in turn co-activate astrocytes [6]. Potentially, chronic glial activation alters tau biology, encouraging tangle formation, and diminishes neuronal fitness [4–6]. Furthermore, glial cells may also be physically contributing to the spread of tau pathology [7]. This review summarizes the literature pertaining to the effects of neuroinflammation and gliosis on tau pathology and vice versa. Both in vitro and in vivo mechanistic studies are discussed alongside evidence from neuropathology and neuroimaging reports in AD and non-AD tauopathy patients. We further explore potential mechanisms by which inflammatory processes may alter the neurodegenerative process.

### Tau pathobiology

The MAPT gene on chromosome 17q21.31 encodes the tau protein which can be alternatively spliced into six distinct isoforms [8, 9]. These isoforms differ by inclusion of either three or four conserved repetitive domains (termed R) as well as the addition of one or two amino-terminal inserts [8]. Aggregates composed of both 3R and 4R tau are seen in disease states, though curiously several tauopathies including PSP, CBD, FTDP-17, and AGD exclusively feature 4R tau deposits. 4R tau does display a higher propensity for aggregation [10] which has led some to postulate that it is more pathogenic [11–13]. A recent study reported significantly higher levels of aggregated hyperphosphorylated tau (p-tau) and less-soluble tau species after using anti-sense oligonucleotides to increase the ratio of 4R:3R tau in mice expressing human tau under the endogenous promoter [12]. While this data supports 4R tau pathogenicity, other factors still contribute to 3R tau aggregation which is seen exclusively in PiD and in the mixed 3R and 4R tau inclusions in AD and CTE [1, 2].

In a healthy brain, tau is predominantly localized in mature neuronal axons and primarily functions to promote microtubule assembly and stability as well as vesicle and organelle transport along microtubules [14–16]. Phosphorylation of serine and threonine residues flanking the microtubule binding domain of tau regulate its interactions with tubulin and influence its conformational state [2, 17]. Therefore, inappropriate phosphorylation of tau at these regions can lower its affinity for tubulin and inhibit its ability to promote microtubule assembly [15]. Free tau species are vulnerable to hyperphosphorylation, which can leave the intrinsically disordered protein more prone to forming β–sheet conformations that promote aggregation into filamentous neurofibrillary tangles (NFTs) that fill neuronal soma and dense neuropil threads (NTs) that line neuronal processes. In addition to phosphorylation, tau can undergo a variety of other post-translational modifications such as acetylation [18–20], glycosylation [21, 22], methylation [23, 24], nitration [25, 26], O-glycosylation [27–29], polyisomerisation [30, 31], SUMOylation [32, 33], truncation [34–37], and ubiquitination [38–40]. These modifications alter tau structure, function and cellular localization which influence its pathophysiology [2, 14].

Seminal studies by Braak and Braak first described a spatial and temporal pattern in the appearance of tangles in AD patient brains that follow neuronal networks and correlate with cognitive decline. In the AD pattern, NFTs first appear in the transentorhinal region and progress along anatomical pathways to the hippocampus and eventually the neocortex [41, 42]. An analogous pattern has been recapitulated in two independent mouse models, rTgECtau mice, where mutant tau was exclusively expressed in the entorhinal cortex and neurons containing aggregates but lacking tau mRNA were found downstream in the dentate gyrus and hippocampus [43, 44]. Similar temporal progression of tau pathology is observed in AGD, though the brain regions involved differ [45]. Likewise, the spatial distribution of tangles is distinct in other tauopathies [1, 2] indicating additional mechanisms involving the vulnerability of certain neuronal populations contribute to disease.

The formation of NFTs was once solely attributed to inherent susceptibility of individual neurons to the disease process. While still a contentious topic [46], there is now substantial evidence that also supports the idea that propagation of pathological tau species occurs between cells [47–50]. Tau, not inside a membrane compartment, is readily detected in the conditioned media of cultured neurons [51–57] and in the interstitial fluid (ISF) of the brain under normal conditions [58–61]. The mechanism of tau release is still unclear, though reports have linked it to synaptic activity [54, 59]. It has also been found in exosomes [7, 55, 62]. Once released tau may be taken up by cells via macropinocytosis as well as potentially other mechanisms [56, 62, 63]. How tau escapes endosomal compartments once internalized is unknown, however cell culture studies have demonstrated that misfolded tau aggregates can mediate a templated misfolding or “seeding” of normal, monomeric tau to induce intracellular tau aggregation [57, 64–66]. Indeed, high molecular weight species of tau isolated from the ISF or cerebral spinal fluid (CSF) of transgenic mice or AD patients has been shown to seed intracellular tau both in vitro and in vivo [67, 68]. This emerging data suggests tau secretion may be a physiological process that is hijacked in disease states. In vivo tau spreading models further support this potential mechanism of tau propagation in that injection of recombinant tau fibrils or brain lysate containing tau aggregates into the brains of wildtype or young transgenic mice can induce robust pathology at the site of injection and in anatomically connected regions [69–73]. The induction of tau pathology in mice that do not otherwise develop tau inclusions supports the concept of seeding and the propagation of tau aggregates to neuronal populations anatomically connected to the site of injection supports a non-cell autonomous mechanism for disease progression. However, whether the spread of pathological tau species is necessary or sufficient for tauopathy and neurodegeneration in humans remains to be proven.

Although tau is predominantly produced by neurons in the brain, it is expressed at low levels in oligodendrocytes and astrocytes and tau pathology is prevalent in these cells across tauopathies [74]. Tau accumulates to form fine, branching coiled bodies and argyrophilic threads that line myelinating processes in oligodendrocytes [75, 76]. Astrocytic tau pathology occurs in several tauopathies but can appear differently. Diffuse granular p-tau clustered around a nucleus of dense tangles illustrates tufted astrocytes specific to PSP. Alternatively, circular tau puncta localized to distal processes compose astrocytic plaques in CBD while ramified bushy astrocytes are typical to AGD. Thorn-shaped astrocytes feature perinuclear tau deposits and are relatively more common as they are observed in PSP, AGD, PiD, AD and in the brains of the cognitively normal elderly [74, 77, 78]. The diversity of astrocytic tau pathology and the implications of each subtype are still largely unknown as is the percentage of tau in glial inclusions that are derived from astrocytes and oligodendrocytes versus neurons. Interestingly, glial fibrillary acidic protein (GFAP), an astrocyte-specific marker commonly up-regulated in activated states, is redistributed differently per each astrocytic phenotype [79]. In addition, reactive gliosis correlates more closely with thorn-shaped astrocytes as opposed to tufted astrocytes [77, 78] suggesting the first may be a common pathological response while the latter independent of the reactive gliotic process [74]. Furthermore, tau lesions impact glial functions leading to an array of deleterious consequences both within the glia themselves as well as non-cell autonomous effects on neuronal health. Tau inclusions have also been reported in microglia [80–82] despite a lack of tau expression, providing further evidence that pathological tau may also be transferring between cells in the brain.

### Risk factors for tauopathies implicate a role for gliosis and neuroinflammation

Reactive gliosis and neuroinflammation were historically considered secondary events in tauopathies and other neurodegenerative diseases. Since the start of the twenty-first century however, accumulating evidence has suggested that aberrant activation of microglia and astrocytes drives chronic neuroinflammation which negatively impacts disease progression. Genetic studies have further implicated roles for the innate immune system in neurodegenerative diseases, particularly AD.

Whole exome sequencing studies have identified numerous gene variants that influence risk for developing AD with varying degrees. Notably, variants of TREM2, an immunoglobulin-like cell-surface receptor primarily expressed on microglia in the brain, were recently found to confer a 2 to 4-fold increased risk for AD [83]. Exactly how TREM2 variants confer AD risk is still under investigation, but current studies indicate it may be due to a loss-of-function in lipid sensing, microglia proliferation and or microglial response to Aβ plaques [84]. However, TREM2’s effect on AD risk is still second to the greatest risk factor for late-onset sporadic AD, apolipoprotein E (ApoE). In the brain, ApoE is predominantly secreted by glial cells and functions as a major transporter of lipoproteins between cells in the brain. Of the three ApoE alleles, ε2, ε3, and ε4, the ApoEε4 allele is associated with a 4–12-fold increased risk based on allele dosage [85, 86]. ApoEε4 is largely thought to influence AD pathogenesis by decreasing Aβ turnover and clearance as well as by directly influencing Aβ aggregation [87]. Additionally, ApoEε4 has been found to have reduced ability to suppress inflammatory stimuli and higher densities of NFTs have been reported in ApoEε4 carriers [88]. Interestingly, the ApoEε4 genotype has also been found to be over-represented in FTD [89, 90] including correlating with increased brain atrophy in patients [91]. Therefore, one possibility is that ApoEε4may increase neuroinflammation which may enhance tau pathology and/or neurodegeneration independent of its influences on Aβ. The exact contributions of ApoE and TREM2 on tau pathogenesis remains unclear and should be more thoroughly assessed in future research. Other gene variants associated with influencing AD risk that impact microglia function and complement signaling include CD33, CR1, ABCA7, SHIP1, BIN1, CD2AP, CLU, HLA-DRB5/DRB1, INPP5D, SORL1, EPHA1, PLD3, PICALM, and MS4A [2, 92, 93]. While in some cases protein products of these genes have been found to influence Aβ accumulation and structure such as CD33 and CLU [94, 95], additional studies are needed to understand their implications in primary tauopathies.

In addition to genetic predisposition, there is evidence that environmental factors that promote neuroinflammation contribute to tau pathogenesis. It is widely recognized that traumatic brain injury (TBI) predisposes individuals to dementia, particularly AD. Increasing evidence further indicates that repetitive mild TBI, with or without concussions, can have long-term consequences leading to tauopathy and neurodegeneration as seen in CTE [96]. Neuroinflammation may be a significant contributor to secondary cell death immediately following moderate to severe TBI and inflammatory effects have been shown to persist up to 17 years post-injury [97, 98]. Similarly, mild TBIs instigate reactive gliosis and prime microglia to over-react to future insults. It is hypothesized that while acute gliosis is arguably protective following TBI, repetitive insults provoke microglia and astrocytes to release markedly higher levels of proinflammatory molecules that can affect neuronal homeostasis and regulate tau release and aggregation [99]. Additionally, environmental toxins and viral infections have all been shown activate gliosis and impact tau pathophysiology [100–102]. Altogether, genetic and environmental risk factors for AD and other tauopathies implicate that glial cells and chronic inflammation may have a more active role in the degenerative process than previously thought. In AD, Aβ plaque deposition may initially provoke gliosis [4, 103] while repetitive mild TBIs have been shown to prime microglia and lead to exacerbated inflammatory responses that are speculated to contribute to the development of CTE [99]. The hypothesis that chronic neuroinflammation plays a causal role in neurodegeneration is rapidly changing the way the field approaches disease research.

### Microgliosis in tauopathy

Microglia are the resident immune cells in the brain and have a nuanced role in neuroprotection and maintenance of homeostasis. Yet under pathological conditions microglia become activated and transform into a ramified, branched state. These cells have the capacity to migrate, proliferate and efficiently phagocytose pathogens and cellular debris, including protein aggregates [104]. Furthermore, activated microglia may release a host of proinflammatory cytokines including interleukin (IL) 1-beta (IL-1β), tumor necrosis factor alpha (TNF-α), IL-6, IL-18 and interferon gamma (IFN-γ) as well as produce nitric oxide, reactive oxygen species and many others associated with a neurodegenerative phenotype [105]. Microglia can also take on a state that is believed to promote tissue remodeling and repair through release of anti-inflammatory cytokines like IL-4, transforming growth factor beta (TGF- β), YM1, arginase 1, and IL-10. Both phenotypes have been reported in disease states making their roles in neurodegeneration unclear [105]. For instance, one study found significantly higher levels of IL-1β and TGF- β in the frontal cortex of AD patient brains corresponding with the presence of ramified, activated microglia and increased levels in TNF-α and IL-6. Conversely in PSP, only IL-1β was significantly increased in the substantia nigra and subthalamic nucleus [92, 106]. This highlights several inherent differences between AD and other tauopathies. First, the spatial distribution of neuroinflammation is dependent on the deposition of protein aggregates specific to each disease [107]. Second, increases in TGF- β and other cytokines associated with remodeling and repair are often reported in response to plaque deposition and are hypothesized to be protective against Aβ pathology [108, 109]. This indicates neuroinflammation may arise differently in AD due to Aβ deposition as opposed to pure tauopathies like PSP and CBD where only tau deposits are seen. In contrast, expression of IL-1β, TNF-α and IL-6 all feed into a cascade that leads to increases in tau hyperphosphorylation, reduction in synapse markers, and neuronal loss [110]. Limited data is available regarding cytokine transcript levels in human tissue of other rarer tauopathies, however neuropathologists note morphologically activated glial cells routinely accompany tau deposition [74]. It is possible that both inflammatory and repair associated microglia coexist in disease states as they try to combat the accumulation of misfolded proteins while also attempting to counteract neuroinflammation. Meanwhile, experimental evidence does demonstrate that dysregulation of proinflammatory molecules is detrimental for tau pathology.

Recently, studies have begun using PET to further examine neuroinflammation in the neurodegenerative process. The most popular tracers investigated to date bind to translocator protein (TPSO) which is expressed on activated microglia, astrocytes, and other infiltrating immune cells in the brain. TPSO signal has been shown to increase with microglia activation in tauopathies including AD, PSP, PiD, and FTDP-17 [111–113] as well as several other neurodegenerative diseases and injury models such as other frontotemporal dementias (FTDs), Parkinson’s disease, stroke and TBI [114]. Interestingly, despite many reports characterizing microglial activation in response to Aβ plaque deposition, Maeda et al. observed stronger correlation of TPSO tracer ^11^C–AC-5216 binding in response to NFTs and NTs in AD and non-AD tauopathy brains. These findings were further investigated in PS19 human-tau transgenic mice, which carry a P301S mutation in the human MAPT gene that is causative for FTDP-17 [115]. The authors observed that radiotracer accumulation in the hippocampus and entorhinal cortex strongly correlated to tau deposition. Importantly, TPSO signaling preceded thioflavin-S-positive tangles and MRI-measureable regional atrophy in the mice implying TPSO PET may be more adept at detecting neuroinflammation induced by tau aggregation [111]. Other PET tracers are also being further developed for imaging of neuroinflammation. Future studies are needed to understand how signal changes spatially and temporally relate to pathology progression in human AD and other primary tauopathies. However, these data do support that tau-induced gliosis is an intrinsic process in tauopathies and that further research should aim to elucidate potential causal relationships between microglia activation, tauopathy progression, and neurodegeneration.

### Microglia may contribute to tau spreading

Many groups have reported tau spreading between synaptically connected neuronal populations [43, 44, 64, 69–71], however lateral propagation of tau aggregates has also been described suggesting that multiple mechanisms of spreading may co-exist. In the rTgECtau mice that selectively express human tau in the entorhinal cortex, tau inclusions were seen to propagate to the dentate gyrus and hippocampus by 18 months of age but cortical neurons lacking tau expression outside of the entorhinal cortex did not have tau deposits. Yet by 24 months, tau aggregates were noted in non-synaptically connected neurons as well as glial cells that surrounded degenerating axon terminals [43]. Similarly, Braak staging has also described tau pathology in unconnected brain regions in AD patients past Braak stage III [41] and glial tau pathology is widely observed across tauopathies [74]. Glial cells are potentially affected by tau pathology as neuronal axons and dendrites degenerate and release toxic, aggregated tau species. Additionally, recent work suggests that microglia play a plausible role in the synaptic and non-synaptic spread of tau pathology.

Microglia readily take up both soluble and insoluble forms of tau [7, 56, 82, 116]. Once engulfed, tau is either degraded [116] or re-released in exocytosing microvesicles called exosomes [7, 55]. Interestingly, increased levels of exosome-associated tau have been found by some groups in the CSF and blood of individuals with AD and FTDs [55, 117]. This led one group to hypothesize that microglia actively contribute to tau propagation by phagocytosing and exocytosing tau protein [7]. They observed a significant reduction in pathologically phosphorylated tau staining by AT8 following pharmacological depletion of microglia in two different tauopathy mouse models. Furthermore, microglial ablation rescued neuronal excitability deficits and resulted in significantly lower levels of proinflammatory cytokines. The authors went on to show that microglia rapidly phagocytose tau and secrete it in exosomes. Inhibition of exosome synthesis reduced tau secretion from microglia in vivo and impeded the development of tau pathology. They concluded that microglia play a significant role in non-synaptic tau propagation and neurotoxicity. While intriguing, this study only examined p-tau species, not fibrillar aggregates, and the number of animals used in each experiment was small. In addition, reducing microgliosis and thereby the levels of proinflammatory cytokines may have also altered the progression of tau pathology independent of exosome synthesis. Further work is needed to truly understand the contribution of microglia-derived exosomes in the spread of tauopathy.

### Astrogliosis in tauopathy

Astrocytes are the most abundant cell type in the brain and are instrumental in supporting neuronal health and function. However, astrogliosis can also be a major contributor to chronic neuroinflammation that diminishes neuronal integrity [118]. Many of the proinflammatory cytokines secreted by microglia can also be synthesized and secreted by astrocytes. Furthermore, signals secreted by microglia, such as IL-1β, TNF-α, IL-6, and C1q have been shown to co-activate astrocytes leading to neuronal dysfunction and death [107]. For instance, reduction of astrocyte-derived cytokine S100β was reported in a study that inhibited IL-1R signaling in mice [119]. The authors demonstrated that IL-1β stimulated S100β secretion that activated GSK-3β in neurons to reduce neuronal β-catenin signaling which has been implicated in tau phosphorylation [120]. Yet, blocking IL-1R restored β-catenin levels by inhibiting GSK-3β. This demonstrates that IL-1β has indirect effects on neurons via affecting cross-talk with astrocytes. Interestingly, IL-1β has also been shown to impact inflammatory responses of astrocytes by binding to and stabilizing IL-6 and COX-2 mRNA, which was shown to be dependent on PKC kinase [121]. Recent studies have further demonstrated how inflammatory signaling can regulate toxic gain-of-function and loss-of-function in astrocytes. These studies illustrated activated microglia induce what was termed an “A1” astrocytic subtype by secretion of IL-1α, TNF, and C1q. A1 astrocytes lost their ability to promote neuronal survival, growth, synaptogenesis, and phagocytosis and were also highly toxic to neurons, though the exact mechanism of toxicity is still unknown [122, 123]. Importantly, this group has demonstrated that A1 astrocytes are upregulated in AD and other neurodegenerative disorders, though additional tauopathies were not tested. This study highlighted the array of consequences that microglial-derived cytokines can have on astrocyte gene expression and function which can ultimately impact neuronal integrity.

As previously described in this review, astrocytic tau pathology is common across many tauopathies. Why and how tau accumulates in astrocytes however is still not understood and very little research has attempted to investigate these questions. Tau accumulation in astrocytes has been reported in some murine tauopathy models [124, 125], however there has been no characterization beyond the initial lesions. One study did attempt to investigate consequences of astrocytic tauopathy in mice by placing the wild-type and a mutant P301L human tau transgene under control of an astrocyte specific promoter [126]. These mice, particularly those expressing P301L tau, displayed age-dependent phosphorylation, fibrillization and asymmetric accumulation of tau in astrocytes beginning at 12 months and progressing up to 24 months of age. The astrocytic pathology in the mice resembled both tufted astrocytes, characteristic of PSP, and astrocytic plaques that are more commonly seen in CBD. In addition to tau deposition, astrocytes displayed a redistribution of the cytoskeletal protein GFAP characteristic to human neuropathology in tauopathies [79]. Tau accumulation and GFAP displacement was followed by induction of low molecular weight heat shock proteins as well as mild disruption of the blood brain barrier evidenced by higher levels of immunoglobulin (IgG) and albumin in brain regions with robust pathology. Finally, neurofilament staining revealed ballooned neurons and axonal degeneration in areas with abundant tau pathology [126]. Likewise, expressing tau in either glia or neurons in a *Drosophila* model was neurotoxic and co-expression in both cell types synergistically enhanced cell death [74, 127]. While these are admittedly artificial models of astrocytic tauopathy, they demonstrate that tau accumulation in astrocytes is sufficient to cause neuronal degeneration.

### Secreted proinflammatory factors

#### Interleukin 1 beta (IL-1β)

Arguably the most prominent cytokine consistently upregulated in AD and related tauopathies is IL-1β. This proinflammatory marker is expressed by multiple cell types in the brain, yet pro-IL-1β transcripts are thought to first be synthesized by microglia in response to insult or injury [128, 129]. Pro-IL-1β is cleaved into its bioactive form by interaction with caspase 1 proteases that are activated by inflammasomes. Once released, IL-1β binds to its cognate receptor, the type 1 IL-1β receptor (IL-1R), which is expressed on many cell types throughout the brain including neurons, though generally IL-1β exerts its primary actions on microglia, astrocytes, and endothelial cells. Binding of IL-1β to IL-1R elicits transduction signaling that activates nuclear factor kappa B (NF-kB) and mitogen-activated protein kinase (MAPK) pathways to promote production of itself as well as induce expression of other proinflammatory cytokines like TNFα and IL-6 [129].

In accordance with upregulation of IL-1β transcripts, caspase 1 levels are elevated in cortical and hippocampal AD brain lysate compared to age-matched controls [109]. Fibrillar Aβ has also been shown to activate caspase 1 via the NALP3 inflammasome, leading to the release of IL-1β [130]. One study further investigated genetic deletion of NALP3 or caspase 1 in APP/PS1 mutant mice (expressing mutant form of the amyloid precursor protein and presenilin 1 genes) and found that gene deficiency increased Aβ phagocytosis by microglia which led to a reduction in plaque deposition and protective effects on learning and memory [109]. Likewise, another group reported overexpression of IL-1β enhanced plaque-associated microglia and attenuated Aβ pathology in the 3xTg mouse model [131]. These mice express mutant forms of APP, PS1 and tau and therefore develop both plaque and tangle pathology. These data indicate that IL-1β signaling may be protective in the context of Aβ pathology in AD. In contrast, p-tau was increased in the IL-1β-3xTg mice despite reductions in Aβ plaques [131]. Although there may be confounds due to the concurrent Aβ pathology in the mouse model, this result suggests a detrimental relationship between neuroinflammation and tauopathy.

Both in vitro and in vivo studies have demonstrated that IL-1β signaling mediates tau phosphorylation by multiple kinases and results in synapse loss and neuronal dysfunction. In culture, microglia activated by lipopolysaccharide (LPS) produced high levels of IL-1β and TNF-α that resulted in greater p38-MAPK signaling. This led to increases in p-tau as well as reduced synaptophysin levels in neuron-microglia co-cultures. Analogous effects were observed when cultures were treated with recombinant IL-1β. Importantly, treatment with an IL-1β receptor agonists or an anti-IL-1β antibody attenuated the effects of activated microglia on neuronal tau and synaptophysin, while anti-TNF-α antibodies were ineffective [132]. This demonstrates that activated microglia secrete IL-1β that is critical for enhancing inflammation and prompting neuronal damage via kinase transduction pathways in vitro. Similar increases in p38-MAPK and glycogen synthase kinase 3 (GSK-3β) signaling were also observed in the IL-1β-3xTg mice that displayed higher levels of p-tau [131]. In addition, age-related microglial activation has been reported in Tg4510 human-tau transgenic mice corresponding with the appearance of insoluble tau aggregates. LPS treatment markedly exacerbated glial activation and p-tau in these mice and microglia were observed to cluster in p-tau burdened areas such as the hippocampus, though cell to cell association was rarely observed with tau-positive neurons [133]. Likewise, another group found that LPS-induced microglial activation further exacerbated IL-1β levels and tau hyperphosphorylation in 3xTg mice by activation of cyclin-dependent kinase 5 (cdk5) and formation of a p25 fragment. Administration of the cdk5 inhibitor, roscovitine, markedly blocked tau phosphorylation [134]. In a follow-up study, an IL-1R blocking antibody reduced IL-1β and TNF-α concentrations in 3xTg mice as well as p-tau levels. Suppression of these effects corresponded with significantly reduced p38-MAPK, GSK-3β and cdk5/p25 activity which are kinases known to phosphorylate tau as well as participate in proinflammatory signal cascades [119]. These data suggest that IL-1β can regulate neuronal kinase activity but the precise signaling pathways linking IL-1β to tau phosphorylation cannot be determined without further mechanistic studies. Additional work is also needed to clarify if fibrillar, insoluble tau is affected by IL-1β signaling or if other mechanisms contribute to tangle formation. Most recently, it has been shown that epigenetic changes may account for increases in IL-1β through down regulation of sirtuin 1 deacetylase in aging microglia. It was shown that PS19 tau transgenic mice also have age-dependent deficiency in sirtuin 1 which elevates IL-1β transcription via CpG hypomethylation of the IL-1β promoter. Tau phosphorylation and solubility were not reported in this study, however the authors did show that CpG sites are significantly hypomethylated in blood samples of patients with FTD and PSP compared to cognitively normal controls and that methylation at these sites correlated with increases in IL-1β [135]. Altogether, these studies reveal a critical role for IL-1β in the regulation of neuroinflammation and tau pathogenesis. Future studies utilizing pure tauopathy models and tissue samples from primary tauopathies will help elucidate the specific effects of IL-1β on tau-mediated neurodegeneration.

The interaction between IL-1β driven neuroinflammation and neuronal tau hyperphosphorylation may be partially regulated by the microglial-specific fractalkine receptor (CX3CR1). Genetic deletion of CX3CR1 in mice expressing human tau under its endogenous promoter (htau mice) led to substantially increased microglial activation indicated by CD68 staining as well as elevated levels of p-tau and insoluble tau aggregates [136]. Additionally, p38-MAPK levels were significantly increased in CX3CR1 deficient htau mice, though no changes were detected in GSK-3β or p25. To examine the possibility that CX3CR1 deficient microglia were affecting tau phosphorylation in neurons, the authors placed conditioned media from CX3CR1−/− microglia on wildtype neurons and observed comparable increases in p38-MAPK and p-tau. However, pretreating the neurons with an IL-1R antagonist attenuated the effects of CX3CR1−/− conditioned media, thereby suggesting microglial-derived IL-1β promotes tau phosphorylation via p38-MAPK in neurons [136]. More recently, another group also reported increased microgliosis and IL-1β levels in young CX3CR1 deficient htau mice which appeared to precede tau pathology, accelerate p38-MAPK activation and p-tau accumulation, and result in reduced hippocampal weight in aged mice [137]. These observations corresponded with a reduction in synaptosome associated protein 25, critical for synaptic vesicle fusion, as well as learning and memory deficits. Finally, adoptive transfer of microglia from CX3CR1−/− htau mice into non-transgenic recipients resulted in increased AT8 p-tau staining and p-38-MAPK signaling which was blocked by co-injection with an IL-1R agonist [137]. These results add further credence to the hypothesis that neuroinflammation may accelerate tau pathology by influencing its phosphorylation state, cause neuronal dysfunction, and ultimately lead to neurodegeneration. However, it should be noted that IL-1β is not sufficient to cause neurotoxicity or neurodegeneration in the absence of tau [136, 138]. Additionally, further experiments are needed to truly elucidate if neuroinflammation is sufficient to induce tau seeding or accelerate the spread of tau pathology. Nevertheless, IL-1β is clearly a pivotal cytokine capable of driving chronic gliosis, influencing tauopathy progression and impacting tau-induced neurodegeneration.

#### Tumor necrosis factor alpha (TNF-α)

TNF-α is another proinflammatory marker that has been implicated in neurodegenerative diseases. It is a key immunocytokine known to orchestrate communication between immune cells and control their many functions throughout the body. In the brain, TNF-α is critical for development, physiology, synaptic plasticity, sleep and circadian cycling, and normal behavior [139, 140]. It is expressed at low basal levels, but can be rapidly upregulated in response to injury. TNF-α has two primary receptors, TNFR1 which is constitutively expressed throughout the brain and TNFR2 which is inducible and primarily localized to glial and endothelial cells [141]. Binding of TNFR1 induces apoptotic signaling while TNFR2 activation results in nuclear entry of NF-kB and promotes transcription of pro-survival genes [140, 142]. In disease, TNF-α has been shown to lead to neuronal apoptosis by activation of caspases 1 and 3, overstimulation of glutamate receptors and inhibition of early long term potentiation dependent on p38-MAPK activation. Additionally, induction of the NF-kB pathway by TNF-α stimulates the release of the proinflammatory enzyme cyclooxygenase 2 (COX-2) [140], as well as activation of c-Jun N-terminal kinase (JNK) which has been shown to phosphorylate tau [143]. These pathways and kinases have also been implicated in affecting tau pathophysiology and neuronal dysfunction.

The majority of studies to date have investigated the role of TNF-α in relation to Aβ pathology in AD, yet a few reports also detail effects on tau. Investigation into TNF-α signaling in AD first began when it was found to co-localize with plaques in post-mortem analysis of AD brains [144]. TNF-α levels were also found to be elevated in the CSF of AD patients and correlate with disease progression [145, 146]. Therefore, one group began by investigating the interaction between Aβ and TNF-α signaling. Aβ is capable of binding to TNFR1 which ultimately leads to activation of NF-kB and neuronal apoptosis [147]. Furthermore, overexpression of TNF-α in 3xTg mice led to enhancement of the local inflammatory environment, increased intracellular Aβ levels and tau hyperphosphorylation. These ultimately led to neuronal death marked by a loss of NeuN-positive neurons in the injected region [148]. Conversely, another group found that global knockout of TNFR1 and TNFR2 receptors in 3xTg mice worsened Aβ and tau pathology [149]. This indicates that TNF-α signaling may be important in early disease states or that there were possibly developmental deficits due to the loss of TNFR1 and 2 that led to long term consequences in protein aggregation. Unfortunately, no studies have truly tested the effects of TNF-α signaling in pure tauopathy models despite TNF-α being implicated in activating pathways involved in tau pathogenesis such as caspase 1 as well as p38-MAPK and JNK kinases. One study did combine fluorescence lifetime imaging microscopy with Förster resonance energy transfer techniques (FRET) to study tau aggregation in response to TNF-α stimulus in vitro. They reported that microglial-derived TNF-α was capable of inducing tau aggregation in neurites [150]. Future studies are needed to fully understand the role of TNF-α in tauopathy-driven neurodegeneration and whether it is a viable drug target to slow disease progression.

#### Interleukin 6 (IL-6)

IL-6 is a crucial cytokine for micro- and astrogliosis in the brain conveying paradoxical proinflammatory and neurotrophic effects. It has been shown to support proliferation of both astrocytes and microglia [151, 152] and enhance microglial phagocytosis [153, 154]. Like TNF-α, IL-6 has been found in Aβ plaques and is elevated in the CSF and plasma of AD patients [105]. Interestingly though, increased IL-6 levels have been shown to correlate more closely with NFT burden in AD patients rather than neuritic plaques [155] as well as age-related cognitive decline in humans [156]. In cell culture, Aβ stimulates IL-6 release which leads to microglial differentiation, thought to further enable them to degrade Aβ [154]. In fact, IL-6 overexpression in APP transgenic mice reduced plaque deposition [157]. Conversely, treatment of hippocampal neurons with IL-6 led to tau phosphorylation via cdk5 and p35 [158]. IL-6 can also activate JAK-STAT pathways, NMDA receptors, and p38-MAPK kinases which all have been shown to contribute to p-tau formation [154, 159]. Therefore, IL-6 is another example of how cytokine signaling may prove protective in the context of Aβ pathology yet detrimental for tau. Additional work exploring the influences of IL-6 on the development and spread of tau pathology will help clarify this cytokine’s role in tau-stimulated pathogenesis and degeneration.

#### Complement proteins

The complement system is composed of many proteins that react with one another to opsonize pathogens and signal immune cells in order to combat infectious agents. Activation of the complement cascade is initiated by one of over 30 soluble factors that all can lead to assembly of C3 convertase, which results in C3a and C3b products. These peptides can either signal immune cells to phagocytose opsonized antigens as well as induce cell death [160]. Additionally, complement signaling may lead to a host of other cellular functions including release of proinflammatory cytokines such as IL-1β, TNF-α, IL-6 and IL-18 [161]. Interestingly, C1q has also been shown to interact with protein aggregates including Aβ and tau. A study dating back to 1996 described the localization of C1q with Aβ plaques as well as C1q-positive structures along NFTs in human AD brain tissue [162]. The authors speculated that C1q was binding to extracellular NFTs, though at the time tau was thought to strictly be an intracellular protein. Given current knowledge in the field regarding tau release and propagation, it would be interesting to investigate if C1q may label tau once it is released into the ISF prior to being taken up by another cell. The authors also observed C1q staining along apical dendrites of otherwise apparently healthy neurons. This finding is intriguing in the context of later work that has uncovered a role for complement signaling to mediate synaptic pruning by microglia. While this occurs normally in the developing brain [163, 164], Hong et al. found that C1q was also upregulated prior to plaque formation in multiple mouse models of Aβ pathology and co-localized with synaptic markers. Furthermore, oligomeric Aβ induced C1q deposition while C3 was necessary for oligomeric-Aβ-dependent engulfment of synapses by microglia. Therefore, the authors proposed a model where C1q and Aβ operate to activate the complement cascade and drive synapse elimination by microglia in AD [165]. Interestingly, another recent publication reported C1q deposition was dependent on ApoE isoforms, with aged human ApoEε4 knock-in mice accumulating significantly more C1q in the hippocampus than ApoEε2 mice. This may have interesting implications in the context of AD considering ApoEε4 is the greatest risk factor for late-onset AD dementia. Additionally, ApoEε2 enhanced synapse elimination by astrocytes while ApoEε4 prevented it [166]. Astrocytes are the major source of ApoE in the brain but the implications for impaired astrocyte-mediated synapse phagocytosis require further experiments. However, these studies do suggest that both microglia and astrocytes have important, active roles in disease processes. It will also be interesting to see if future studies reveal a role for complement signaling in mediating synapse loss in primary tauopathies or in aggravating tau pathology and neuronal loss as it has been shown for other proinflammatory molecules.

#### Additional cytokines and factors

There are vast arrays of additional cytokines that are dysregulated in AD and related tauopathies. Interleukins such as IL-18, IL-34, IL-4, IL-10 IL-13, and others have been reported as either up or downregulated in patient brain tissue, CSF or blood [92, 103, 167]. Specifically, increased IL-18 signaling has been shown to activate JNK and p38-MAPK pro-apoptotic pathways [105]. Another study also found that IL-18 may impact hyperphosphorylation of tau via cdk5/p35 and GSK-3β kinases [168]. Meanwhile, anti-inflammatory molecules like IL-4 and IL-10 may antagonize the proinflammatory effects of IL-1β and IL-6 [103]. Other factors such as TGFβ, IFNγ, COX-2, CCL2 and free radicals like reactive oxygen species and nitric oxide have also been implicated in aspects of inflammation, tauopathy and neurodegeneration. For instance, IFNγ signaling has been shown to lead to tau phosphorylation and acceleration of neuritic tangle pathology while TGFβ has been shown to be a key regulator of various microglial factors including CX3CR1 and numerous interleukins [110, 169]. Continued research in these and other aforementioned molecules will illuminate the role of neuroinflammation in tauopathy and neurodegeneration.

### Neuroinflammation in tauopathies: Cause or effect?

Gliosis and neuroinflammation are prevalent in tauopathy patient brains [4, 5, 106] and recapitulated across many animal models [127, 170–174]. Furthermore, microgliosis, astrogliosis and inflammatory markers like IL-β, TNF-α and IL-6 have been shown increase in response to tau pathology [171]. However, it is still a matter of debate whether aberrant neuroinflammation causes tau pathophysiology or if glial cells respond first to tau toxicity. Yoshiyama et al. has provided the most compelling evidence to date that microgliosis can precede tau tangle formation and is capable of driving neurodegeneration. In their initial paper describing the PS19 tau transgenic mouse, the authors were surprised by the striking increase in CD11b immunoreactivity in 3-month-old animals, prior to the accumulation of tau deposits [115]. Additional radiograms utilizing [^3^H]DAA1106 clearly demonstrated an age-dependent microglial-activation in the hippocampus, amygdala and entorhinal cortex. Moreover, CA3 neurons in the hippocampus of 4-month-old mice were immunoreactive for IL-β and COX-2. To further test the hypothesis that microgliosis was capable of driving tauopathy, the immunosuppressant FK506 was given to the mice beginning at 2 months of age. Not only did treatment significantly reduce tau pathology and brain atrophy, it dramatically increased the life-span of the mice. While these data offer a mechanistic link between aberrant microglial activity and tauopathy progression, more recent studies have revealed earlier forms of tau aggregation in PS19 tau transgenic mice using a cellular FRET-based biosensor assay that utilizes recombinant repeat domain tau (RD-tau) fused to either yellow or cyan fluorescent protein. In the presence of tau seeds, the RD-tau aggregates and FRET signal can be measured by flow cytometry [66]. This assay has led to new insights regarding tau toxicity and disease progression. For instance, it has revealed tau seeding activity in PS19 mice as early as 1.5–2 months of age [66]. Therefore, it is possible that tau seeds invoke early microglial activation, which in turn accelerate tau pathology and neurodegeneration. It also raises interesting questions regarding microglial activation and tau seeding, especially considering the dramatic effects of FK506 treatment. Do activated microglia physically contribute to tau seeding or spread? Do proinflammatory molecules activate pathways that encourage tauopathy development? Is gliosis required for neurodegeneration in tauopathies or does it exacerbate it? Future studies should investigate the link between microgliosis, neuroinflammation and tau seeding as well as consider the possible effects of tau strains which have been shown to have different degrees of seeding activity and provoke unique microglial phenotypes [175].

### Implications for therapies

Despite the significant clinical and economic burden tauopathies place on society, there are currently no treatments capable of curing or even slowing disease progression. The pursuit for tau-based therapies has rapidly expanded over the past ten years and today drug discovery efforts are fervently ongoing. Drug development is currently investigating tau immunotherapies, small molecule inhibitors, and microtubule stabilizers [176–179]. There have been many preclinical studies published in these areas and some agents have just started progressing through clinical trials.

While drug discovery is an active area of research in the dementia field, it is also important to have a full understanding of the mechanisms underlying disease. Initial immunization studies for Aβ were halted due to severe neuroinflammatory adverse events, some of which resulted in death. Additionally, many of the Aβ monoclonal antibodies have led to side effects, such as microhemorrages and brain edema, which are attributed to microglia-induced damaged to cerebral vasculature as they respond to antibodies coating cerebral amyloid angiopathy [180]. Therefore, careful consideration of the neuro-immune system should be taken in approaching tau immunotherapy. Some studies have reported that microglia can mediate tau clearance and this is enhanced with tau monoclonal antibody treatment [116, 181]. Antibodies typically stimulate antigen phagocytosis in an Fc-dependent (fragment crystallizable domain) manner. However, binding of Fc receptors is also known to activate microglia and stimulate release of proinflammatory molecules which can exacerbate the degenerative process [182]. Alternatively, it has been shown that antibodies that block tau seeding activity can prevent the formation of tangles and brain atrophy in mice [183]. It is possible that simply binding extracellular tau may prevent propagation of pathological tau seeds and slow disease progression. This type of mechanism would not require Fc effector function for tau antibodies. A recent study reported that an Fc-effectorless tau antibody reduced p-tau in vivo akin to the full-effector antibody with the same epitope specificity and affinity [184]. In addition, the effectorless tau antibody did not stimulate microglial release of IL-1β, TNF-α or IL-6 in vitro, which in the case of the full Fc effector containing antibody led to neuronal toxicity. A recent paper also showed that single chain fragment variables (scFvs) derived from an anti-tau antibody decreased p-tau accumulation in the brain of PS19 tau transgenic mice indicating that microglial activation via the Fc domain of an antibody is not required for the protective effect of such a treatment [185]. Additional work is needed to stringently test if Fc-effectorless tau antibodies can prevent formation of aggregated tau and neurodegeneration in vivo as well as characterize the effects of inflammation stimulated by tau immunotherapies.

There is also renewed interest in targeting inflammatory pathways since the discovery of TREM2-mediated risk for AD. In the past, clinical trials with various NSAIDs and glucocortoroids failed to rescue cognitive deficits in AD patients or prevent disease progression despite promising data from preclinical animal studies [186]. However, it is possible that more targeted therapies or starting earlier in the disease process will have positive effects. Current data indicates that inflammation is initially stimulated by Aβ in AD and that chronic gliosis influences tau pathogenesis. If this is true, it is possible that targeted therapies that interrupt neuroinflammation may be utilized after Aβ accumulation begins to delay or prevent tauopathy in AD. In the context of primary tauopathies, targeting specific proinflammatory molecules or pathways may alter the progression of disease and symptoms.

## Conclusions

**Fig. 1:**
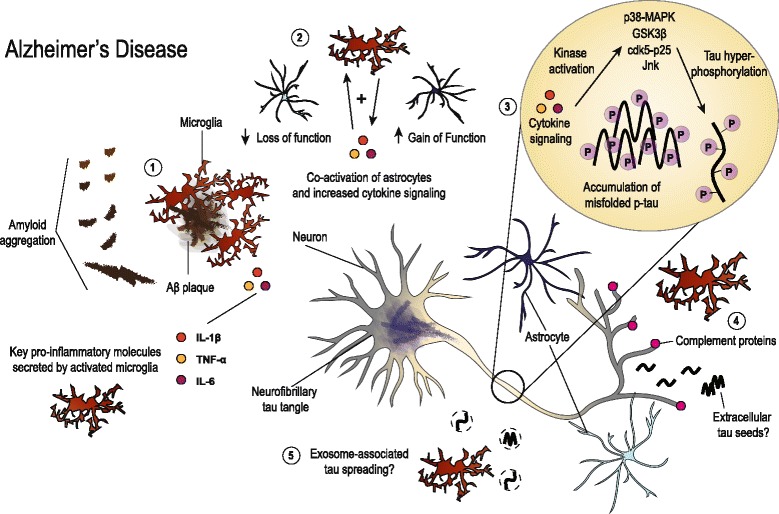
Illustration summarizing the hypothesized roles of gliosis and neuroinflammation in AD. Aggregation of Aβ likely stimulates microglia early in disease and may instigate initial neuroinflammation (1). While gliosis has been shown to be beneficial in reducing plaque burden and mitigating amyloid-associated pathologies, long-term stimulation creates a permissive environment for chronic neuroinflammation. Pro-inflammatory cytokines such as IL-1β, TNF-α and IL-6 further activate microglia and astrocytes, leading to both loss and gain of functions (2). These molecules have been shown to disrupt neuronal homeostasis and alter tau biology. Cytokine signaling has been linked to activation of kinases that phosphorylate tau, which may incite early tau dysfunction and ultimately influence misfolding and accumulation (3). Concurrently, there is deposition of complement proteins at neuronal synapses that can signal microglial pruning and initiate synapse loss (4). Microglia have also been implicated in facilitating the spread of tau via exosomes (5). Additionally, neuronal activity-dependent release of extracellular, misfolded tau may incite neuronal dysfunction or spread of tau pathology along synaptically connected neuronal populations. Ultimately, gliosis and chronic neuroinflammation combine with plaque and tangle pathologies to drive neurodegeneration in AD

Accumulating evidence clearly illustrates a role for gliosis and neuroinflammation in tau pathogenesis and neurodegeneration. However, initiation of inflammatory pathways may occur differently depending on the disease. Fig. 1 summarizes the proposed roles of gliosis and neuroinflammation in AD that haven been discussed in this review. In AD, Aβ aggregation likely stimulates early gliosis and release of inflammatory mediators such as IL-1β and C1q. These molecules may act via autocrine and or paracrine signaling to increase levels of other proinflammatory cytokines such as TNF-α or IL-6 from microglia, astrocytes, neurons, and potentially other cell types in the brain. Early complement signaling has also been shown to stimulate microglial-mediated phagocytosis of synapses. Together these mechanisms may lead to early neuronal dysfunction and synapse loss. Microgliosis can also co-activate astrocytes, provoking both loss and gain of functions impacting neuronal health. Additionally, neuroinflammation offers one way by which tau biology may be altered in AD through increasing phosphorylation that may promote protein misfolding, though other mechanisms likely also co-exist. Tau pathology then progresses through the spread of toxic tau species, neuronal vulnerability, or by combination of both mechanisms. Altogether, Aβ and tau pathology combine with gliosis to drive neurodegeneration and cell death in AD.

**Fig. 2:**
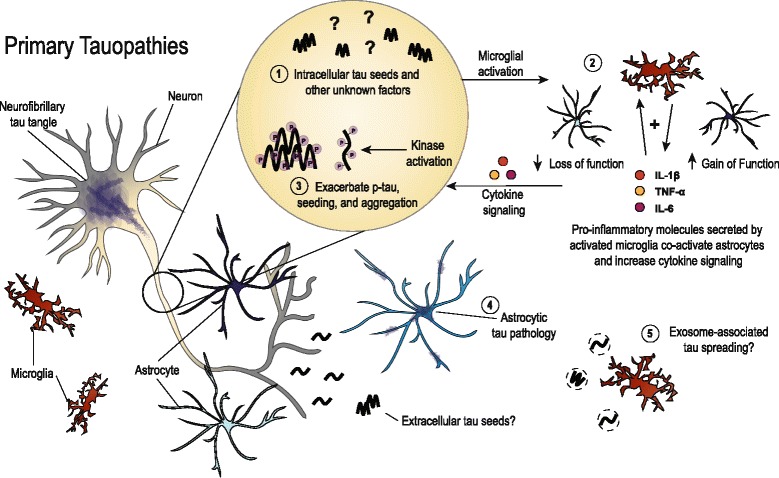
Depiction of the roles that have been described for glial cells in primary tauopathies. In the absence of amyloid pathology, early microgliosis may be initiated by neuronal tau seeds (1), though this remains to be thoroughly tested. Tau seeding has been found early in mouse models of tauopathy, and secreted, extracellular tau also possibly has seeding capability. Either direct or indirect consequences from tau seeds may be responsible for instigating the early microgliosis reported in tau mouse models. Activated microglia then secrete pro-inflammatory cytokines which further exacerbates microgliosis and co-activates astrocytes leading to toxic loss and gain of functions (2). Similar to the role proposed for AD, gliosis and inflammatory signaling can impact tau phosphorylation and possibly enhance misfolding and aggregation (3). In addition, astrocytic tau pathology characterizes several primary tauopathies such as PSP and CBD (4), though the functional consequences of the different aggregate phenotypes that are observed remain unknown. Spread of toxic tau species via microglial-associated exosomes is also a possible mechanism in primary tauopathies (5). Together, chronic neuroinflammation combined with tau pathology diminishes neuronal health and worsens neurodegeneration

Primary tauopathies share many common features with AD, but early mechanisms of neuroinflammation in disease may differ due to the lack of amyloid pathology in pure tauopathies. Fig. 2 illustrates current thinking regarding the role of glial cells in these diseases, though there is a need for more literature directly pertaining to tau-mediated neurodegenerative mechanisms outside of the AD field. Microgliosis may be sparked by early tau aggregates, possibly tau seeds, in primary tauopathies. This may then initiate a positive feedback loop, similar to that for AD, which amplifies microglial activation, co-activates astrocytes, and aggravates pathways influencing tau hyperphosphorylation and aggregation. Tau accumulation in glial cells leads to further dysfunction that impacts neuronal viability in a non-cell-autonomous fashion, However, it remains unclear why there are phenotypic differences in the brain regions affected by tauopathy and the types of tau aggregates that have been described in neuropathology studies. Together, tau pathology and neuroinflammation synergistically drive neurodegeneration and clinical symptoms in tauopathies such as PSP, CBD, FTDP-17, PiD, AGD and CTE.

Clearly tau pathogenesis significantly contributes to neurodegenerative diseases. However, there are many outstanding questions that require further research and clarification. For instance, do the genetic risk variants recently found to associate with AD play a role in primary tauopathies? Certain features are shared between AD and other diseases that feature tau aggregation, but there are also some clear distinctions. Therefore, it is also necessary to understand how neuroinflammatory mechanisms, such as cytokine and complement signaling, function in AD and in the absence of amyloid pathology. Furthermore, the influence of these signaling pathways should be explored further, beyond tau phosphorylation, to truly understand if neuroinflammation can contribute to the formation of insoluble, fibrillar tau aggregates. In extension, it is unclear what, if any, role gliosis plays in neurodegeneration. Is gliosis required for brain degenerative phenotypes or are neuroinflammatory molecules released from activated glial cells the main contributors? It may be that tau aggregates are the spark needed for cellular dysfunction in the brain, and neuroinflammation the accelerant for disease progression. Additionally, the phenotypic differences in astrocytic tau pathology are intriguing and the functional consequences should be further explored. Finally, recent evidence has indicated that microglia may be contributing to the spread of tau aggregates. The extent to which microglia may physically contribute to disease progression or be influencing tau seeding or spread via neuroinflammation needs further investigation. Addressing these questions will ultimately help explain the relationship between microglial activation, tauopathy progression, and neurodegeneration and hopefully facilitate the creation of drugs that will be effective in treating tauopathy disorders.

## Abbreviations

AD: Alzheimer’s disease
AGD: Argyrophilic grain disease
ApoE: Apolipoprotein E
Aβ: Amyloid beta
CBD: Corticobasal degeneration
cdk5: Cyclin-dependent kinase 5
CSF: Cerebral spinal fluid
CTE: Chronic traumatic encephalopathy
CX3CR1: Microglial-specific fractalkine receptor
Fc: Fragment crystallizable
FRET: Förster resonance energy transfer
FTDP-17: Frontotemporal dementia with parkinsonism linked to chromosome 17
GFAP: Glial fibrillary acidic protein
GSK-3β: Glycogen synthase kinase 3
IFNγ: Interferon gamma
IL: Interleukin
IL-1β: Interleukin 1 beta
ISF: Interstitial fluid
JNK: c-Jun N-terminal kinase
MAPK: Mitogen activated protein kinase
NF-κB: NF-kappaB
NFT: Neurofibrillary tangle
NT: Neuropil thread
p-tau: Phosphorylated tau
PSP: Progressive supranuclear palsy
TGF-β: Transformation growth factor beta
TNF-α: Tumor necrosis factor alpha
TPSO: Translocator protein
